# A Comparative Study of Land Cover Classification by Using Multispectral and Texture Data

**DOI:** 10.1155/2016/8797438

**Published:** 2016-06-08

**Authors:** Salman Qadri, Dost Muhammad Khan, Farooq Ahmad, Syed Furqan Qadri, Masroor Ellahi Babar, Muhammad Shahid, Muzammil Ul-Rehman, Abdul Razzaq, Syed Shah Muhammad, Muhammad Fahad, Sarfraz Ahmad, Muhammad Tariq Pervez, Nasir Naveed, Naeem Aslam, Mutiullah Jamil, Ejaz Ahmad Rehmani, Nazir Ahmad, Naeem Akhtar Khan

**Affiliations:** ^1^Department of CS & IT, The Islamia University of Bahawalpur, Punjab 63100, Pakistan; ^2^Department of Computer Sciences, CIIT Lahore, Punjab 54000, Pakistan; ^3^Key Laboratory of Photo-Electronic Imaging Technology and System, School of Computer Science, Beijing Institute of Technology (BIT), Beijing 100081, China; ^4^Department of Bioinformatics and Computational Biology, Virtual University of Pakistan, Lahore, Punjab 54000, Pakistan; ^5^Department of CS, NFC IET, Multan, Punjab 60000, Pakistan; ^6^Department of CS, Virtual University of Pakistan, Lahore, Punjab 54000, Pakistan; ^7^Faculty of Information Technology, University of Central Punjab (UCP), Lahore 54000, Pakistan

## Abstract

The main objective of this study is to find out the importance of machine vision approach for the classification of five types of land cover data such as bare land, desert rangeland, green pasture, fertile cultivated land, and Sutlej river land. A novel spectra-statistical framework is designed to classify the subjective land cover data types accurately. Multispectral data of these land covers were acquired by using a handheld device named multispectral radiometer in the form of five spectral bands (blue, green, red, near infrared, and shortwave infrared) while texture data were acquired with a digital camera by the transformation of acquired images into 229 texture features for each image. The most discriminant 30 features of each image were obtained by integrating the three statistical features selection techniques such as Fisher, Probability of Error plus Average Correlation, and Mutual Information (*F* + PA + MI). Selected texture data clustering was verified by nonlinear discriminant analysis while linear discriminant analysis approach was applied for multispectral data. For classification, the texture and multispectral data were deployed to artificial neural network (ANN:* n*-class). By implementing a cross validation method (80-20), we received an accuracy of 91.332% for texture data and 96.40% for multispectral data, respectively.

## 1. Introduction

Image processing and remote sensing are playing a vital role for the betterment of the agriculture field [1]. By using this technology, we can classify vast land cover area into different categories [2]. Not only would this be helpful for the socioeconomic sector but also it fulfills the needs of the future for sustainable development. In the twenty-first century, the world is facing the challenge of hunger, food, and poverty [3]. This issue can be resolved by increase in crop production and better utilization of cultivated land. Land cover information is necessary for different policy making, planning, and management purposes including land record of a forest, desert, farmland, and wetland as well as other biophysical resources, which are required for land cover information. Researchers are trying to get the benefits of technology by involving it in the agriculture field [4]. It is being tried to enhance the cultivated land area and monitor the land through field survey [5]. For the success of such surveys, more time with expensive labor is required. In developing countries like Pakistan, it seems to be very difficult to spend a lot of resources on such projects. Whether directly or indirectly, almost 50% of the population of these countries is associated with the agriculture profession [6]. All the preceding issues highlight the importance of the proper land management and better crop growth and production. According to geographical distribution of the country, it is categorized into different land cover types like barren, fertile, rocky and sandy, and so forth. In Pakistan, the conventional field based survey system could not be properly managed due to financial and technical limitations. For this reason, remote sensing technology could not be used for natural resource management up till now, as was proposed by the relevant professionals [7]. Many researchers used this technology for better resource management; for example, a two-layer conditional random field (CRF) model was proposed for land cover and land use classification [8]. Similarly, a multilayer conditional random field (MCRF) land classification model was suggested. It was used for multitemporal with multiscale remote sensing data [9]. A gray level cooccurrence matrix with different window size images was used to find the four land types of aerial data. Different statistical features, that is, dissimilarity, homogeneity, angular second moment, and entropy, were calculated to classify the data [10]. A supervised pixel-based classification algorithm was used by implementing Markov Random Field (MRF) technique to distinguish the agriculture land cover area (cropland and grassland). It gave the satisfactory results for updating in GIS database for the cropland and grassland region [11]. A new idea of image spectroscopy (IS) and near-infrared spectroscopy (NIRS) was presented by [12] and it predicted that in the future it will be potentially used in many disciplines like geology, environmental sciences, precision agriculture, urban development, water and soil sciences, and so forth. The objective of this study is to design a simple, concise, and robust framework to classify the five types of land cover data in an absolute natural environment by using spectral and texture features. To accomplish this study, the procedural steps of data collection, image preprocessing, feature extraction, feature selection, feature reduction, and classification are employed for this classification framework.

## 2. Study Area

**Figure 1:**
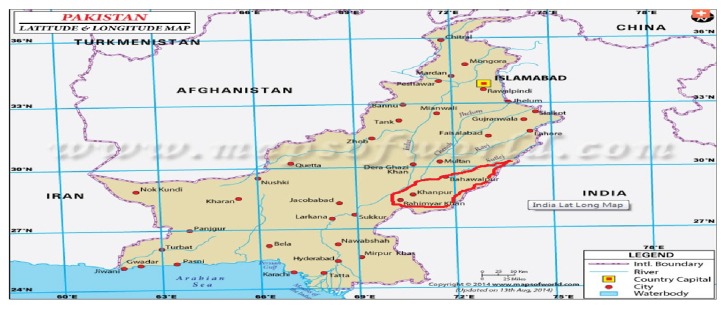
Geographic location of the study area. The red highlighted left side on the map represents the study area [13].

In this study, involving the technologies, that is, image processing and remote sensing, in land cover classification instead of conventional field surveys is tried. This study is conducted at division Bahawalpur of Punjab province (Pakistan) and covered area is 45,588 square kilometers, which is the areawise largest division of this province, located at 29°23′44′′N and 71°41′1′′E and shown in Figure 1. This study focuses on the land cover assessment, management, and classification through photographic and multispectral radiometric data of this area, which is mostly barren and desert rangeland. It will also help to monitor the land cover changes and estimate the biomass of land vegetation, which is used for forecasting different crops yield assessment.

## 3. Material and Methods

In this study, two types of data are being acquired: (1) photographic data for texture features and (2) radiometric data for remote sensing. Remote sensing data are acquired by using a device named multispectral radiometer (MSR5), CROPSCAN. It is a handheld device, which provides data equivalent to satellite Landsat 5 TM (Thematic Mapper). MSR5 provides an alternative way of acquiring data for remote sensing where satellite or radar datasets are not easily available. Its output data comprises five spectral bands which include visible (blue, green, and red) and infrared and shortwave infrared ranges from 450 nm to 1750 nm, whereas photographic data are acquired by a digital camera. This study will be based on the analysis of five types of land cover datasets, bare land, desert rangeland, fertile cultivated land, green pasture, and Sutlej river land.

### 3.1. Photographic Data and Image Acquisition

**Figure 2:**
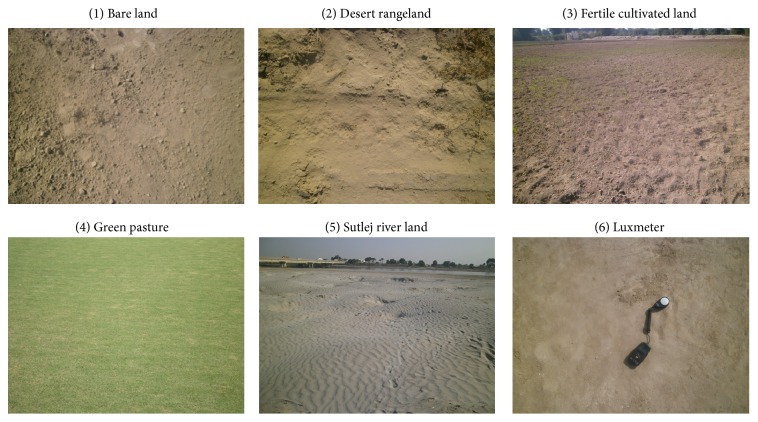
Five land cover images and Luxmeter.

The abovementioned five different types of land cover plots have a 43560-square-foot area (1 acre) for each type. Digital photographs of bare land, desert rangeland, fertile cultivated land, green pasture, and Sutlej river land are taken by a digital Nikon camera, model COOLPIX having a 10.1-megapixel resolution, which are shown in Figure 2. The 15 colored images of each type of land cover with the dimensions of 4288 × 3216 pixels and 24-bit depth of jpg format are acquired. To increase the dataset, four nonoverlapping regions of interests (ROIs) of size (512 × 512) on each image are developed; in this way total (75 × 4 = 300) subimages data are arranged for the analysis. The photographic data are acquired at the altitude of 5 feet from the ground surface of the same specific location where radiometric data are acquired.

To keep away from the sun shadow effect, the data are acquired at noontime (1.00 pm to 3.00 pm) under a clear sky. At the time of data acquisition, the light intensity is measured by digital Luxmeter MS 6610, MATECH, and described in Table 1.

### 3.2. Remote Sensing Data Acquisition

Remote sensing can be defined as the collection of data in the form of radiations about an object taken from a particular distance [16]. Remote sensing is now playing an important role in many disciplines, that is, environmental sciences, geography, agriculture, forestry, botany, meteorology, oceanography, and earth sciences [17].

### 3.3. Multispectral Radiometer (MSR5)

Multispectral radiometer (MSR5) is made up by CROPSCAN Inc. (USA) for data collection. MSR5 has the quality to provide data similar to satellite Landsat 5 TM. CROPSCAN MSR5 has been already used for the assessment and measurement of crops weeds effect [18] and vegetation cover estimation and diseases estimation [19, 20]. For remote sensing, data are acquired 50 MSR scans of each plot at 5 feet's height of land cover surface. Each MSR5 scan contains five wave bands, three visible (blue, green, and red) and two invisible (near infrared and shortwave infrared). Five different types of land cover contain total 250 spectral data instances.

### 3.4. Spectral Features

Multispectral radiometer (MSR5) has five different sections of spectrum, including visible, which include the blue, green, red, near infrared (NIR), and shortwave infrared (SWIR) [15]. MSR5 spectrum consists of different wavelengths, which are measured in nanometer (nm) and described in Table 2.

**Figure 3:**
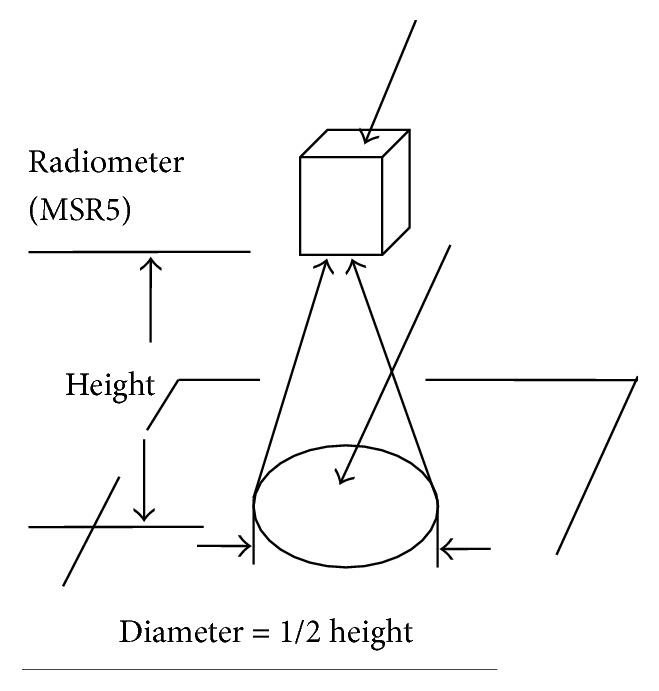
MSR5 data acquiring process for each scan [14].

This device will be used to collect data at a specific height normal to the land surface. The device that is used in this study is MSR5 with serial number 566. It contains five spectral bands, which are shown in Table 2. In this study, the data acquired by this device in each scan is at the height of 5 feet and it covers land area for each scan that is almost half of the under height which is almost 2.5 square feet's diameter of land cover. The multispectral data acquiring process is shown in Figure 3.

### 3.5. Proposed Methodology

**Figure 4:**
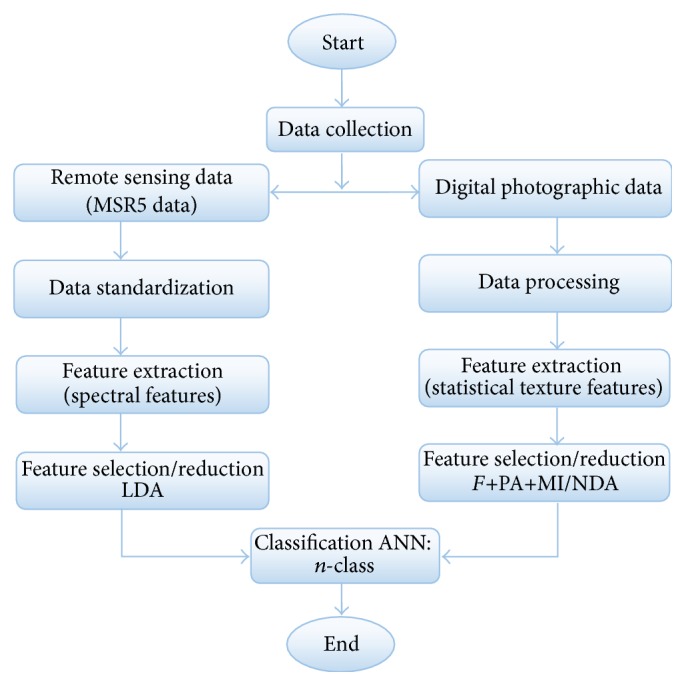
Proposed spectra-statistical design framework for land cover classification.

For this study any special laboratory setup for morphological and color features has not been established, just acquired texture features for photographic data and spectral features for remote sensing MSR5 data. A novel spectra-statistical design framework is proposed for subjective land cover classification. The proposed framework is described in Figure 4.

The proposed spectra-statistical design framework describes the functionality of this study that is given below in detail. The proposed methodology has been implemented by using MaZda software version 4.6 on Intel® Core i3 processor 2.4 gigahertz (GHz) with a 64-bit operating system.

### 3.6. Preprocessing

Each image has a vast irrelevant area, so prior to further processing the relevant portion of the image was extracted. The extracted relevant portions of the images were converted to grayscale images (8 bits) and were stored in bitmap (bmp) format because the software MaZda better works for this format to calculate the statistical texture parameters [21]. By using image converter software, the contrast of grayscale images was enhanced.

### 3.7. Feature Extraction

Transition of an image into its statistical attributes is called feature extraction, which are used for the classification of an image. There are different methods for feature extraction, that is, texture, Gabor, wavelet transform and boundary features, and so forth.

### 3.8. Texture Features

Statistical texture features are categorized into the first order, which relates to the intensity of the individual pixels, while the second order relates to the occurrence of neighboring pixels. First-order statistical parameters are directly based on histogram features of an image while second-order parameters are based on the gray level cooccurrence matrix (GLCM). For this study, total 229 statistical texture features are calculated for each region of interest (ROI) by using MaZda software version 4.6. The calculated parameters are grouped as 9 first-order statistical parameters and 11 second-order (Haralick) statistical parameters derived from GLCM in all four directions (0°, 45°, 90°, and 135°) up to 5-pixel distance 220 (11 × 4 × 5) [22]. It means that each region of interest (ROI) has presented by 229 statistical textural features. Statistically total 300 subimages' data are presented by a 300 × 229 = 68700 dimensional features' vector space.

### 3.9. Feature Selection

Feature selection is an important study area where hundred to thousand features space datasets are available. Its objective is to select the most significant features in the employed procedures. Furthermore, reliable classification results are based on a large number of features; usually big data have been required, which is not easily available. It is necessary to reduce the dimensionality of statistical features vector space, which has the capability to discriminate and classify the different types of these land cover classes. These approaches have been used for the selection of the most discriminant set of features. Finally, we can achieve fast and cost-effective classification accuracy based on these selected features. In this study, three features selection approaches, that is, Fisher Coefficient (*F*), Probability of Error (POE) plus Average Correlation Coefficient (ACC), and Mutual Information (MI) Coefficient, have been used to reduce the features vector space. In this study, features selection has been performed through the combined set of the three already mentioned approaches (*F* + PA + MI) for the entire features vector space by using MaZda software. Fisher Coefficient (*F*) [23] mathematically is described as

(1) FK2M2=1/1−∑a=1aPa2∑a=1a·∑j=1aPaPjLa−Lj2∑a=1a·PaMa2,

where *K* is between-class variance, *M* is within-class variance, *P*_*a*_ is probability of feature *a*, and *M*_*a*_ and *L*_*a*_ are variance and mean value of feature *a* in the given class.

Probability of Error plus Average Correlation Coefficient (POE + ACC) [24] is defined as

(2) POEfj=number of misclassified samplestotal number of samples,f2=fj:minimumjPOEfj+correlationf1,fj,fn=fj:minimumjPOEfj+1k−1∑a=0k−1·correlationfa,fj.

Mutual Information (MI) Coefficient [25] is explained by the given mathematical relation:

(3) $$\begin{array}{c} I\left(\;F,C\;\right)=\sum f\sum cP\left(\;F\cdot C\;\right)\log2\frac{P\left(F\cdot C\right)}{P\left(F\right)P\left(C\right)}. \end{array}$$

It is important to show here that, for this study, as all the 229 calculated features of each image have not been equally significant for land cover classification, MaZda software selects 10 most discriminant features for each method in descending order according to their significance. For analysis, it is observed that combined set of feature selection approaches provide better classification results; in this way total 30 features (10 features by each approach) have been selected [26]. A set of 30 features has been acquired for further processing. These selected features have been shown with respect to their corresponding three feature selection techniques including* F*, PA, and MI in Table 3.

No doubt, in Table 3, the MI based selected features are highly correlated such as “inverse difference moment” but they have different interpixel distance and direction and due to this difference, their calculated values are also different. For each pixel distance (*d*) and angular direction value (*θ*), the intensive nature of computation is involved and acquired different texture feature values for the same parameter, that is, “inverse difference moment.” For this study, we have taken *d* = 1-, 2-, 3-, 4-, and 5-pixel distance with angle *θ* = 0°, 45°, 90°, and 135°. Therefore, for this reason, we cannot ignore any value of the given texture features. Each value (MI base texture features) actually describes the land cover dataset into its own dimension or direction and as a whole these features reveal the entire texture patterns. It is reported by different researchers [10, 11] that five different control features such as window size, texture derivative(s), input channel (i.e., spectral channel to measure the texture), quantization level of output channel (8 bits, 16 bits, and 32 bits), and the spatial components (i.e., interpixel distance and angle during cooccurrence matrix computation) play a vital role during the analysis of GLCM texture features.

### 3.10. Feature Reduction

Feature reduction techniques are also called feature projection. In feature reduction, the original feature space of selected features is transformed to a new space having lower dimensionality. It is also called projection space in which data are clustered in respective classes. These feature projection techniques include the linear discriminant analysis (LDA), principal component analysis (PCA), and nonlinear discriminant analysis (NDA). For such purpose, usually PCA, LDA, and NDA approaches are employed. Features reduction techniques maintain the actual structure of the data as much as possible while reducing the number of dimensions. Thus in the reduced feature space, the execution time with cost is also reduced and we get smaller dimension space. It is observed that the obtained results are approximately reliable to the original data space. Before starting the classification, the data are standardized to reduce the impact of undesirable variation within the data due to exceptions and other factors by applying the following statistical equation:

(4) $$\begin{array}{c} K_{i}^{\prime }=\frac{K_{i}-\overset{-}{K}}{\sigma }, \end{array}$$

where *K*_*i*_′ is the consistent value of the *i*th feature and *i* = 1, 2, 3,…, *n*. *K*_*i*_ is original feature value, $\overset{-}{K}$ is mean feature value, and *σ* is standard deviation.

The above discussed feature selection techniques (*F* + PA + MI) only select the significant features but do not quantify how much these can be classified. To get the feature data projection, the selected 30 features' data are deployed to nonlinear discriminant analysis (NDA) available in B11 software integrated with MaZda [27]. In this technique there are 3 layers (input layer and the first and second hidden layer and output layer) of processing elements (neurons) that are presented. NDA can be described by logistic function. Its value is equal to 0.5 for *α* = 0, and it changes smoothly from 0 to 1 for *α* varying from large negative to large positive values:

(5) $$\begin{array}{c} \left(\;\alpha \;\right)=\frac{1}{1+\exp\left(-\alpha \right)}. \end{array}$$

If *X* is the feature vector and it is the input to the artificial neural network (ANN), the input terminals are equal to *N*_*x*_. Vector *Y* is the output of ANN, whose dimension *N*_*y*_ is equal to the number of types in the dataset. Thus, the ANN had *N*_*y*_ output terminals:

(6) $$\begin{array}{c} Y_{n}=\left[\;U_{n0}+\overset{N_{g}}{\underset{k=1}{\sum }}U_{nk}g_{k}\;\right]. \end{array}$$

Now, here *n* = 1, 2, 3,…, *N*_*y*_. Consider

(7) $$\begin{array}{c} g_{k}=\left[\;V_{k0}+\overset{N_{h}}{\underset{j=1}{\sum }}V_{kj}h\;\right]. \end{array}$$

Here *k* = 1, 2, 3,…, *N*_*g*_. Consider

(8) $$\begin{array}{c} h_{j}=\left[\;W_{j0}+\overset{N_{x}}{\underset{i=1}{\sum }}{W_{ji}X}_{i}\;\right]. \end{array}$$

Here *j* = 1, 2, 3,…, *N*_*h*_.

Supervised learning methods are based on input patterns and correct classes where they belong to {*x*_*i*_, *d*_*i*_}, where *i* = 1, 2, 3,…, *M*. For this purpose, the following errors function is calculated:

(9) $$\begin{array}{c} E=\frac{1}{2}\overset{M}{\underset{i=1}{\sum }}\overset{Ny}{\underset{n=1}{\sum }}{\left(\;d_{in}-Y_{in}\left(\;X_{i};U,V,W\;\right)\;\right)}^{2}. \end{array}$$

While for MSR5 datasets, linear discriminant analysis (LDA) gives the better results for features data clustered and projection. Let *x*_*i*_^(*k*)^ denote the *i*th pattern in class *i*, where *i* = 1, 2, 3,…, *M*_*k*_, and *k* = 1, 2, 3,…, *N*_*c*_. Define the within-class scatter matrix *C*_*W*_ as

(10) $$\begin{array}{c} C_{W}=\frac{1}{M}\overset{N_{c}}{\underset{k=1}{\sum }}\cdot \overset{M_{k}}{\underset{i=0}{\sum }}\cdot \left(\;X_{i}^{k}-U^{k}\;\right){\left(\;X_{i}^{k}-U^{k}\;\right)}^{t}, \end{array}$$

where *M*_*k*_ is the mean vector of class *k*. Similarly, define the between-class scatter matrix *C*_*B*_ as

(11) $$\begin{array}{c} C_{B}=\frac{1}{M}\overset{N_{c}}{\underset{k=1}{\sum }}M_{k}\cdot \left(\;U^{k}-U\;\right){\left(\;U^{k}-U\;\right)}^{t}. \end{array}$$

Here *M* is the mean vector of the shared data. The total scatter matrix is the objective of LDA and through this we can get a linear transform matrix:

(12) $$\begin{array}{c} C_{t}=\frac{1}{M}\overset{N_{c}}{\underset{k=1}{\sum }}\cdot \overset{M_{k}}{\underset{i=0}{\sum }}\cdot \left(\;X_{i}^{k}-U\;\right){\left(\;X_{i}^{k}-U\;\right)}^{t}. \end{array}$$

The proposed NDA architecture is given for both types of dataset in Tables 4 and 5.

### 3.11. Classification

**Figure 5:**
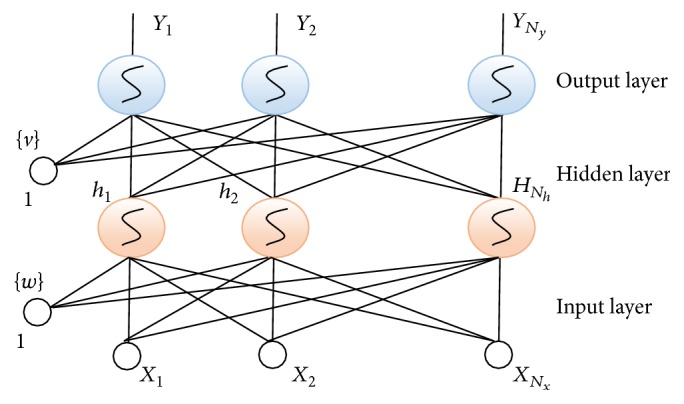
Implemented ANN classifier model [15].

For this work, we have applied supervised classification artificial neural network (ANN). This classifier is employed due to two reasons; first of all we have supervised data (due to five land covers) and it is discussed by [28] that ANN is a strong and efficient technique for noisy data and also for those datasets which are acquired in natural open environment. The implemented classifier based on feed forward approach with a single hidden layer of sigmoidal neurons is shown in Figure 5. If *x* is the number of deployed input feature vectors to ANN classifier then input terminals are equal to *N*_*x*_. The output feature vector is *y*, whose dimensions *N*_*y*_ are determined by the number of classes to be classified. Thus, the ANN has *N*_*y*_ output terminals:

(13) $$\begin{array}{c} Y_{k}=\left[\;V_{k0}+\overset{N_{h}}{\underset{j=1}{\sum }}V_{kj}h_{j}\;\right], \end{array}$$

where *k* = 1, 2, 3,…, *N*_*y*_ and the outputs of the hidden layers are given as

(14) $$\begin{array}{c} h_{j}=\left[\;W_{j0}+\overset{N_{x}}{\underset{i=1}{\sum }}W_{ji}X_{i}\;\right]. \end{array}$$

We see here that *j* = 1, 2, 3,…, *N*_*h*_.

For training and testing purpose, the weight coefficients are adjusted and how much actual output value *y* is close to the desired output *d* is observed.

Supervised training techniques are based on input patterns and correct categories where they belong to {*x*_*i*_, *d*_*i*_}, where *i* = 1, 2, 3,…, *M*; then following is the error function which is reduced by changing of weights *v* and *w*:

(15) $$\begin{array}{c} E=\frac{1}{2}\overset{M}{\underset{i=1}{\sum }}\overset{N_{y}}{\underset{n=1}{\sum }}{\left(\;d_{in}-Y_{in}\left(\;X_{i};V,W\;\right)\;\right)}^{2}. \end{array}$$

## 4. Results and Discussion

### 4.1. Photographic Data

For photographic dataset, the first attempt for features data selection and reduction is performed by individual feature selection techniques like Fisher (*F*), Probability of Error plus Average Correlation Coefficient (POE + ACC), and Mutual Information (MI) techniques on the basis of ROIs (64 × 64), (128 × 128), (256 × 256), and (512 × 512). Now the selected features are deployed for raw data analysis (RDA), principal component analysis (PCA), linear discriminant analysis (LDA), and nonlinear discriminant analysis (NDA) projection spaces to verify the capability of data clustering. Here, better data clustering based on the NDA approach has been received as compared to the other three approaches. It is observed that the discussed above first three ROIs do not give satisfactory results. They have received less than 70% feature projection accuracy based on these three ROIs, which are not acceptable, whereas, for ROI (512 × 512), we received 80%, 84%, and 88.324% accuracy by using* F*, PA, and MI, respectively, in projection space of NDA. Because it has been reported by a number of researchers that usually the classification is proportional to the number of features deployed [28], the same strategy was implemented to have better results. For this purpose, the authors merged the selected features by the already above discussed three approaches (*F* + PA + MI). In this way, a set of 30 features (10 features of each selection method) is received by combining these three approaches on ROI (512 × 512). Then these 30 features were deployed to RDA, PCA, LDA, and NDA by using the* K*-fold (80-20) cross validation method. It has been observed that NDA has given better data clustering and projection accuracy 99.64% as compared to the other three features reduction techniques. These results are summarized in Table 6.

**Figure 6:**
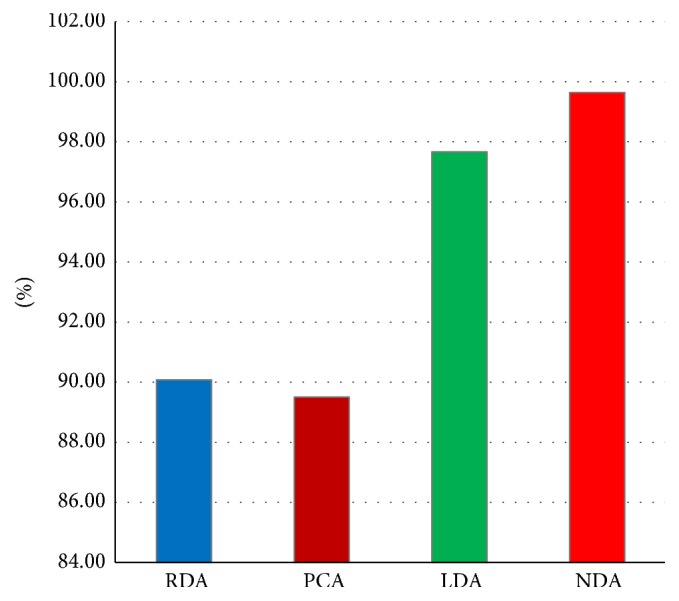
Digital photographic features data projection graph.

The statistical texture data analyses of RDA, PCA, LDA, and NDA are shown in Table 6. From this table, it is clear that NDA leads the best data projection accuracy of 99.64% as compared to the remaining three approaches including RDA, PCA, and LDA.  Figure 6 represents the photographic features data clustering of five input land cover classes in NDA projection space.

**Figure 7:**
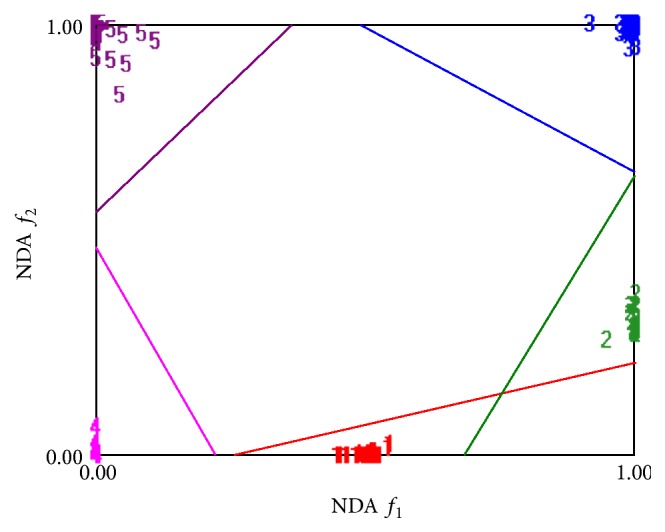
Statistical texture features data clustered results for NDA.

It is observed by different researchers [27, 28] that feature reduction techniques including raw data analysis (RDA), linear discriminant analysis (LDA), and principal component analysis (PCA) performed well on linearly separable data because PCA and LDA use linear transformation of the input data. These techniques have the ability for feature compression. The most expressive features (MEF) are obtained by PCA and the most discriminating features (MDF) are found from LDA technique. These features vectors have not as many features as the original feature vectors space does. Due to this reason, they cannot help in classification of linearly nonseparable data. Such data need hypersurfaces instead of hyperplanes for data clusters separation. That is why nonlinear discriminant analysis (NDA) is used for nonlinear transformation of the feature vectors, such that the input data are projected on a space (probably of lower dimensionality as compared to PCA and LDA) in which they become linearly separated. In this study, this technique is implemented by using a feed forward artificial neural network (ANN) with two hidden layers of sigmoid-type neurons. To verify the capability of data clustering based on selected features of complex nonlinear datasets, nonlinear discriminant analysis (NDA) is the best approach. Therefore, we have employed the same approach; moreover, B11 software has also a number of options by which NDA may be configured to have the best result. Nonlinear discriminant analysis (NDA) graph shows the properly clustered data into its five appropriate classes. Data clustered graph is shown in Figure 7.

By the implementation of (ANN:* n*-class) training and testing, available in B11 integrated with MaZda software, is performed to verify the validity of classifier. For this purpose, a cross validation* K*-fold (80-20) method is used. For training purpose, 48 data instances of each ROIs size (512 × 512) from land cover type are used. Total 240 data instances out of 300 were used for training with each iteration. Testing is performed on 60 data instances (12 data instances from each land cover type). Here an accuracy of 100% is acquired when the classifier is trained over the architecture setting already discussed above in Section 3.11 and an average classification accuracy of 91.334% is obtained when the classifier is tested for photographic data. So, five types of land cover data are classified properly by using (ANN:* n*-class) method. Statistical texture data are shown in Table 7.

The performances of the classifier in testing phase for different classes are summarized in confusion matrix, Table 8. Total 300 data instances of photographic data (60 data instances of each land cover) are shown in the appropriate five different classes.

**Figure 8:**
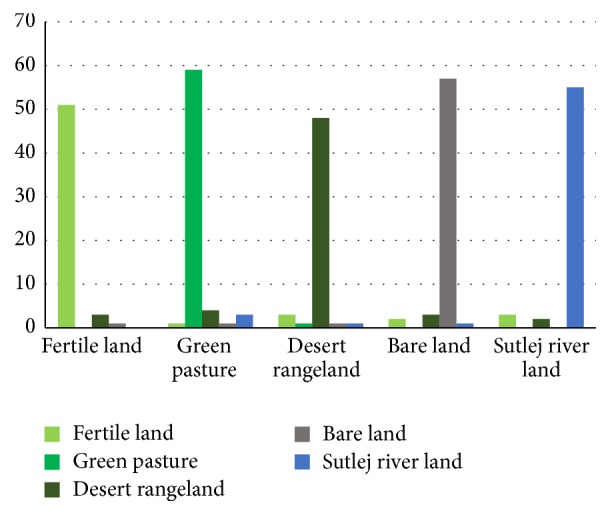
Confusion graph for statistical texture test data classification.

Here confusion matrix, Table 8, for photographic data is presented of five different land cover types by graphical way in Figure 8.

### 4.2. Spectral Data

As we have already mentioned, a scene is completely explored based on five spectral bands, blue, green, and red, near infrared, and shortwave infrared acquired by MSR5. The whole data (250 scans) are acquired by MSR5 and deployed to RDA, PCA, LDA, and NDA to verify the validity of data projection. Now, data projection accuracy of 98.7% for RDA, 98.4% for PCA, 99.5% for LDA, and 99.4% for NDA is received. It is clear that the best feature projection accuracy is received by LDA approach as shown in Table 9. The results of data projection are presented in Table 9 in detail.

**Figure 9:**
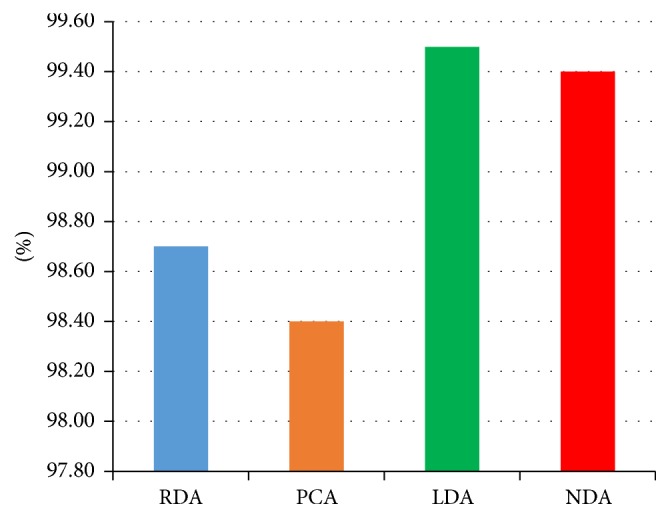
Multispectral feature data projection graph.

Multispectral features data analyses of RDA, PCA, LDA, and NDA are shown in Table 9; this shows that LDA outperforms the others and gives 99.5% feature data projection accuracy. For feature reduction techniques, feature data projection graph of MSR5 is shown in Figure 9.

**Figure 10:**
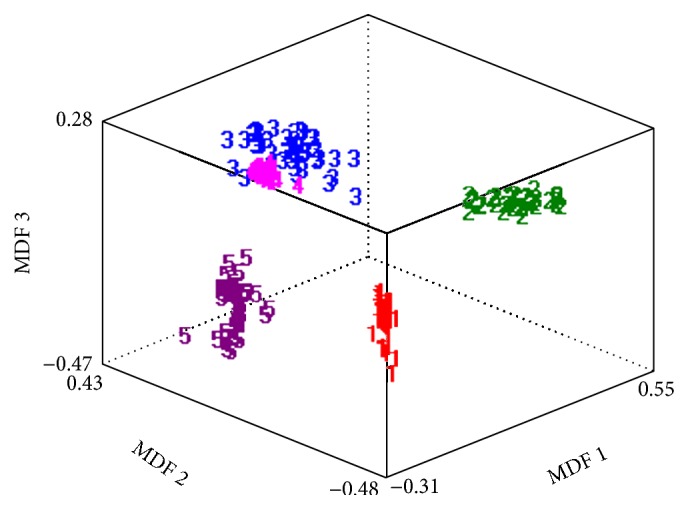
Multispectral features data clustered result for LDA.

Linear discriminant analysis (LDA) graph shows the properly clustered data into its five appropriate classes as compared to other employed reduction techniques. Data cluster graph is shown in Figure 10.

**Figure 11:**
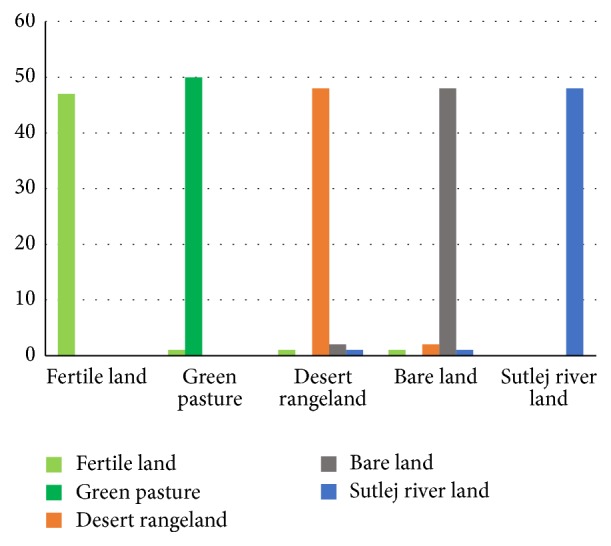
Confusion graph for multispectral test data classification.

For the purpose of training and testing, artificial neural network (ANN:* n*-class) classifier has been employed; the same* K*-fold (80-20) cross validation method is also used for multispectral data classification. A dataset of two hundred scans of five multispectral parameters, blue, green, and red, near infrared, and shortwave infrared, is deployed for (ANN:* n*-class) training purpose with the same architecture settings as mentioned above in Section 3.11. The output training results for multispectral data are summarized in Table 10 and are represented graphically in Figure 11. Similarly, under the same architecture setting as discussed earlier in classification Section 3.11, ANN classifier is tested by deploying 50 disjoints data instances (10 data instances of each land cover type) of the selected five multispectral features of land cover types. MSR5 data are shown in Table 10.

ANN classifier revealed very promising results during this training and testing phase. An average classification accuracy of 100% has been achieved when the classifier has been trained over this data. Similarly, an average classification accuracy of 96.40% has been achieved when classifier is tested. So, five land cover types' data are classified properly by using (ANN:* n*-class) methods. Confusion matrix table of multispectral data classification by using (ANN:* n*-class) method of five different land cover types is shown in Table 11.

Now, confusion matrix graph for MSR5 data is presented by using the (ANN:* n*-class) method of five different land cover types which are shown in Figure 11.

**Figure 12:**
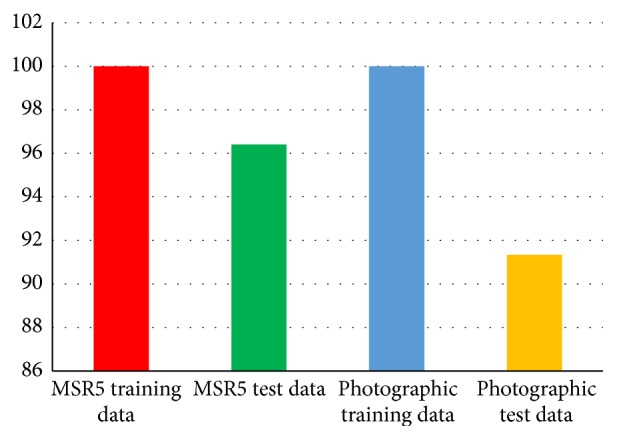
Multispectral and statistical texture data graph.

When comparing both multispectral and statistical texture data classification accuracies, it is observed that multispectral accuracy result is better 96.40% as compared to statistical texture data result which is 91.334%. A comparison graph between multispectral and statistical texture data is shown in Figure 12.

The reason for this classification accuracy difference is that statistical analysis outperforms other methods on fine texture as compared to coarse texture. This is the reason texture data classification accuracy is lower than multispectral data [29, 30]. In this study, the photographic data are taken at 5 feet's height so the areas under these photographs are not equally covered and distributed; besides these ROIs also play an important role for classification [31]; as ROIs size increased then accuracy is also observed better. It is the fact that if photographs are taken on more height and area under the region is covered maximum then classification accuracy can be improved. Secondly it is observed that almost (5% to 6%) better classification results are obtained by the remote sensing MSR5 data as compared to photographic data (400 nm to 700 nm) because MSR5 data comprises visible (400 nm to 700 nm) and invisible near-infrared (NIR) and shortwave infrared (SWIR) (790 nm to 1750 nm) wavelength. Data acquisition techniques with normalization and standardization of data with classifier may also impact on results for better classification. By implementing these sophisticated quantitative parameters rather than conventional qualitative parameters, they can accurately classify the different types of land cover data. Generally, the proposed methodology provides a novel technique for mapping and classifying land cover data by using multispectral and digital photographic data.

## 5. Conclusions

In this study, five types of land cover data are classified by using quantitative parameters instead of conventional qualitative parameters and an accuracy of 96.40% for spectral dataset and 91.334% for statistical texture dataset is achieved. Up to what extent these classes may be classified into their appropriate patterns classes is a difficult task and it is also a verification of intra- and interclassification pattern features of these five land cover data types. Five spectral and nine first-order with eleven second-order cooccurrence matrix features are used to test the land cover datasets which made this framework novel and more reliable and robust than other land classification frameworks in which morphological, size, color, and other geometry features have been used. Artificial neural network is used very effectively for the classification of these five different land cover types such as fertile cultivated land, green pasture, desert rangeland, bare land, and Sutlej river land. In the future, we may enhance this study for hyperspectral data of crop growth and yield assessment. We can also take results with new technique of data fusion by combining MSR5 data with digital photographic data for considering different environmental factors like rain, usage of fertilizers, dry weather, and soil and air moisture effects.

## Figures and Tables

**Table 1:** Time and sunlight intensity information.

Sr. number	Land cover type	Time	Sunshine intensity
(1)	Bare land	1.00 pm	34300 Lux
(2)	Desert rangeland	2.00 pm	34000 Lux
(3)	Fertile cultivated land	1.30 pm	34500 Lux
(4)	Green pasture	1.30 pm	35000 Lux
(5)	Sutlej river land	1.00 pm	34300 Lux

**Table 2:** MSR5 (S. number 566).

MSR types	Blue	Green	Red	Near infrared	Shortwave infrared
MSR5 (generic)	450–520 nm	520–600 nm	630–690 nm	760–930 nm	1550–1750 nm
MSR5 (S. number 566)	485 nm	560 nm	660 nm	830 nm	1650 nm

**Table 3:** Feature selection table (*F* + PA + MI) for ROIs (512 × 512).

Features		
1	*F*	*S*(0,3) correlation
2		*S*(0,4) correlation
3		*S*(0,3) contrast
4		*S*(0,4) contrast
5		*S*(0,5) correlation
6		*S*(0,5) contrast
7		*S*(2,2) correlation
8		*S*(0,3) sum variance
9		*S*(0,1) inv. diff. mom.
10		*S*(0,4) sum variance

11	PA	Percent .01%
12		*S*(1,1) sum variance
13		*S*(0,1) ang. sec. mom
14		Skewness
15		*S*(0,2) sum variance
16		*S*(5,5) entropy
17		*S*(5,−5) inv. diff. mom.
18		*S*(1,0) sum. average
19		*S*(1,0) correlation
20		*S*(3,3) entropy

21	MI	*S*(0,5) inv. diff. mom.
22		*S*(5,−5) inv. diff. mom.
23		*S*(0,4) inv. diff. mom.
24		*S*(4,−4) inv. diff. mom.
25		*S*(0,3) inv. diff. mom.
26		*S*(3,−3) inv. diff. mom.
27		*S*(0,2) inv. diff. mom.
28		*S*(2,2) inv. diff. mom.
29		*S*(2,−2) inv. diff. mom.
30		*S*(0,1) inv. diff. mom.

**Table 4:** NDA architecture for statistical texture dataset.

Input layers = 5	1st hidden layer = 5	2nd hidden layer = 2
Learning rate eta = 0.25	Back propagation iteration = 200000	Optimized iteration limit = 70

*Output layers = 5*		

**Table 5:** NDA architecture for multispectral dataset.

Input layers = 5	1st hidden layer = 5	2nd hidden layer = 2
Learning rate eta = 0.20	Back propagation iteration = 200000	Optimized iteration limit = 70

*Output layers = 5*		

**Table 6:** Statistical texture features data projection table.

Statistical data analysis *K*-fold (80-20)	RDA	PCA	LDA	NDA
1-fold	92.5%	92.50%	97.50%	99.5%
2-fold	88.75%	87.92%	96.25%	100%
3-fold	90%	89.17%	98.75%	99%
4-fold	88.75%	87.50%	96.67%	100%
5-fold	90.42%	90.42%	99.17%	99.69%
*Average accuracy*	*90.08%*	*89.502%*	*97.668%*	*99.64%*

**Table 7:** Classification table of statistical texture data using artificial neural network (ANN: *n*-class).

Statistical data iteration (80-20)	Training dataset	Training data classification accuracy %	Test dataset	Misclassified data	Test data classification accuracy %
1-fold	240	100%	60	5/60	91.67%
2-fold	240	100%	60	6/60	90%
3-fold	240	100%	60	6/60	90%
4-fold	240	100%	60	3/60	95%
5-fold	240	100%	60	6/60	90%
*Average training data classification accuracy: 100%*			*Average test data classification accuracy: 91.334%*		

**Table 8:** Confusion matrix for statistical texture data classification using (ANN: *n*-class).

Type	Fertile land	Green pasture	Desert rangeland	Bare land	Sutlej river land	Total
Fertile land	51	1	3	2	3	60
Green pasture	0	59	1	0	0	60
Desert rangeland	3	4	48	3	2	60
Bare land	1	1	1	57	0	60
Sutlej river land	0	3	1	1	55	60

**Table 9:** Multispectral features data projection table.

Spectral data analysis (80-20)	RDA	PCA	LDA	NDA
1-fold	99%	97.5%	99.5%	100%
2-fold	99%	99%	100%	99%
3-fold	98.5%	98.5%	100%	100%
4-fold	98.5%	98.5%	99%	99%
5-fold	98.5%	98.5%	99%	99%
*Average projection data accuracy*	*98.7%*	*98.4%*	*99.5%*	*99.4%*

**Table 10:** Classification table for multispectral data using artificial neural network (ANN: *n*-class).

Multispectral data iteration (80-20)	Training dataset	Training data classification accuracy %	Test dataset	Misclassified data	Test data classification accuracy %
1-fold	200	100%	50	6/50	88%
2-fold	200	100%	50	2/50	96%
3-fold	200	100%	50	0/50	100%
4-fold	200	100%	50	1/50	98%
5-fold	200	100%	50	0/50	100%

*Average multispectral training data classification accuracy: (100 + 100 + 100 + 100 + 100)/5 = 100%*					
*Average multispectral test data classification accuracy: (88 + 96 + 100 + 98 + 100)/5 = 96.40%*					

**Table 11:** Confusion matrix for multispectral data classification using (ANN: *n*-class).

Type	Fertile land	Green pasture	Desert rangeland	Bare land	Sutlej river land	Total
Fertile land	47	1	1	1	0	50
Green pasture	0	50	0	0	0	50
Desert rangeland	0	0	48	2	0	50
Bare land	0	0	2	48	0	50
Sutlej river land	0	0	1	1	48	50
